# A mechanism to derive more truthful willingness to accept values for renewable energy systems

**DOI:** 10.1016/j.heliyon.2018.e00503

**Published:** 2018-01-09

**Authors:** Mehrshad Radmehr, Ken Willis, Hugh Metcalf

**Affiliations:** a Cyprus International University Business School, Nicosia, Via Mersin 10, North Cyprus; b Centre for Research in Environmental Appraisal & Management, Newcastle University, Newcastle upon Tyne, NE1 7RU, UK; c Newcastle University Business School, 5 Barrack Road, Newcastle upon Tyne, NE1 4SE, UK

**Keywords:** Energy, Economics, Psychology

## Abstract

This paper examines and compares households’ willingness to accept (WTA)/willingness to pay (WTP) ratio for solar power equipment on their premises through both a novel experimental approach and conventional techniques. The experimental approach was administered by using a Becker-DeGroot-Marschak method and cheap talk, with open-ended questions of WTA/WTP. The results were quite striking. The ratio for the incentivised approach was 1.08:1; whereas for the conventional approach it was 3.5:1. The findings suggest that the hypothesis that WTP equals WTA cannot be rejected for the incentivised mechanism, and it appears to control for the individual’s strategic behaviour bias as a treatment against over-estimating WTA and under-estimating WTP. The findings also provide some policy implications for Northern Cyprus: the government can set lower financial incentives to increase the solar power installed capacity on the island.

## 1 Introduction

In 2005 the share of energy from renewable resources in gross final consumption in Cyprus was 2.9%. The European Union (EU) Commission Directive 2009/28/EC established a common framework for the use of energy from renewable resources in order to limit greenhouse gas (GHG) emissions. The EU set an obligatory target for each member state. The EU target for the renewable energy sector’s share of gross final energy use in Cyprus is 13% by 2020.

This target necessitates developing plans to implement renewable energy technology projects and policies in the electricity sector. The North Cyprus government has also set an incentive strategy to expand renewable energy exploitation in an attempt to support environmental improvements; and to have greater self-sufficiency in power generation, as a substitute to the imported sources, whilst maximizing the efficiency of renewable energy sources (RES) utilization.

Cyprus has 300 days of sunny weather per year. There is thus a high potential for solar energy utilization, particularly micro-generation solar panels. The government is attempting to raise people awareness about the benefits of energy efficiency, the need for diversification of sources of energy, and reduced dependence on imported fossil fuels. The aim is to change people’s behaviour towards renewable energy production and consumption, primarily by the use of incentives. The adoption of renewable energy by households is both a private and public good. The public good aspect of renewable energy adoption is the household’s contribution towards reducing carbon emissions and reducing global climate warming. The private good externality element is the reduction in visual amenity of the property as a result of the installation of renewable energy solar panel systems.

The aim of this research is to assess people’s willingness-to-pay (WTP) for micro-generation photovoltaic (PV) systems in Northern Cyprus, and their willingness-to-accept (WTA) compensation to forego their right to micro-generation of PV on their property.

## 2 Theory

CV has been used to measure the monetary value of both gains and losses in the quantity of a good. WTA measures the minimum amount that an individual is willing to accept as just compensation for the loss, whilst WTP measures the maximum amount an individual is willing to pay rather than forego the environmental gain (Hanemann, 1991).

Numerous studies have used CV to measure WTA compensation for the loss, and WTP for the gain, of a ‘public good’, and also private goods, in environmental economics (McFadden, 1994; Carson, 1997; Bateman et al., 2002; Haab and McConnell, 2002; Bateman and Willis, 1999).

The loss of an environmental good is typically valued more highly than an equivalent gain in the good. This asymmetry between willingness-to-accept (WTA) compensation for the loss of a good and WTP for a gain has long been a feature of most CV results. Bishop et al. (1983) explained the discrepancy as an anomaly arising from people’s unawareness of the value of environmental assets and non-market values in monetary terms. Explanations for WTP–WTA asymmetry, in terms of economic theory, have emphasised the role of substitution and income effects (Hanemann, 1991, Hanemann, 1999). Disposable income constrains demand for environmental improvements in terms of WTP, but not WTA compensation; whilst the unique character (low substitutability) of some public and private goods implies high compensation to offset utility loss. However, Plott and Zeiler (2005) using a Becker-DeGroot-Marschak auction mechanism, and respondent or subject training, found no difference between WTP and WTA across a variety of goods, thus calling into question loss aversion theory in real market conditions.

Horowitz and McConnell (2002) analysed 45 studies which reported WTA and WTP values, and found the mean WTA/WTP ratio was approximately 7.0; with a higher WTA/WTP ratio (10.4:1) for public and non-market goods, and a ratio of 2.9:1 for ordinary private goods, with the lowest ratio being for experiments involving forms of money. Haab and McConnell (2002) suggested that the proportion of the difference between WTA and WTP for private goods, such as pens and mugs, is not the same as for public goods, and this notion challenges Hanemann’s assumption based on neoclassical theory. Nevertheless, studies have shown that even for private goods, there is a divergence between WTP and WTA (Horowitz and McConnell, 2002). According to neoclassical theory, the income constraint is a factor limiting the value of WTP. Unlike WTP, WTA is not constrained by income because consumers are able to demand greater monetary amounts.

In survey instruments different methodical biases have been observed by CV practitioners. Sudgen (1999) suggested the use of a well-designed instrument to facilitate the minimisation of these biases to elicit true preferences in accordance with an incentive-compatible mechanism. In attempting to weaken the endowment effect, Plott and Zeiler (2005) proposed the need to control subjects’ misconceptions. This effect can be controlled by using an incentive compatible elicitation mechanism to clarify the minimum WTA and maximum WTP terminologies. Bjornstad et al. (1997) proposed a teaching mechanism to simplify the CV technique, on the basis that the parametric and non-parametric results which suggested that the impact of “learning design” on eliminating of hypothetical bias is highly effective. In addition to teaching and clarification tools, assuring respondents’ confidentiality is an effective tool to control the subject’s misconceptions. Furthermore, research has identified the role of an incentive compatible survey design in eliciting truthful answers in which respondents must view their responses as an effective element over actions or decisions (Carson and Groves, 2007). Potential hypothetical bias can be controlled by clarifying for the respondents what is meant by a minimum WTA and maximum WTP. Indeed, the importance of a market-like environmental setting for a decreasing ratio was recommended nearly thirty years ago by Brookshire and Coursey (1987).

Various mechanisms exist to elicit truthful answers directly, such as take-it-or-leave it offers, Vickrey auctions, nth-price auctions, BDM auctions, and incentive compatible stated preference methods such as contingent valuation and choice experiments. Neill et al. (1994) designed open-ended CVM questions and used two types of hypothetical and second-bid or Vickrey auction surveys to value the same good. In the Vickrey auction, individuals were asked to make the real payment from their own pockets, for the good in question, in order to generate true results. The values from both the hypothetical and Vickrey auctions were compared and the findings indicated that an open-ended hypothetical valuation is not always capable of providing unbiased true values. However, the Vickrey auction’s WTP values were lower than the hypothetical ones, and the values were closer to the real economic values. This suggests that situating individuals in a real market setting supports the use of incentive compatibility in a survey to elicit truthful answers. Similarly, Berry et al. (2012) compared the BDM and take-it-or-leave it values of WTP for clean drinking water technology in northern Ghana. The take-it-or-leave-it survey results showed a higher WTP compared with the BDM. The gap was explained as a possibility the result of strategic behaviour and anchoring effects.

The study reported here uses a Becker-DeGroot-Marschak (BDM) incentive compatible experimental method along with cheap talk to mitigate some of the behavioral and hypothetical anomalies that can potentially affect the use of CV in estimating the benefit of environmental policies. In what follows we present and compare the results of a conventional approach with a BDM experimental approach. The experimental approach shows a lower WTA/WTP ratio than has previously been reported in the environmental economics literature. In addition, the average WTA value was significantly influenced by incentivised BDM setting which sharply reduced the WTA values. Whereas, the average value of WTP was not substantially greater than in the conventional study.

## 3 Background

The high capital cost of micro-generation solar technology is a barrier to accelerating the distribution and supply of the technology. However, consumers can be influenced by financial incentives to install solar panels on their premises. Previous studies have pointed to the viability of grid connected micro-generation solar systems in the residential sector. Scarpa and Willis (2010) suggested that, in the UK, government grants would need to be increased to attract more households to install micro-generation systems and offset the higher cost of the renewable energy (RE) micro-generation systems. However, their results showed that despite households’ enthusiasm for investing and their willingness to pay for micro-generation systems, the benefit households received from micro-generation was not sufficiently large to cover the capital cost of micro-generation energy technologies. Claudy et al. (2011) reviewed the Irish WTP for micro-generation technologies, and found that their WTP was considerably lower than the actual market prices. The main obstacle was said to be the initial cost of purchasing or installation, but they also suggested more market based finance options for consumers such as leasing and ‘fee for service.’ An alternative to leasing and fee for service might be a network connection. Grid connection has a number of advantages over a stand-alone or off-grid system, and may increase the number of investors. It offers both reliability and financial benefits for consumers and an unfailing connection to electricity would be guaranteed. Any excess generated electricity can be exported and sold to the grid and electricity outages can be prevented by importing when there is no sun. In addition, it saves the extra cost of installing batteries. However, although the need for financial incentives to induce consumers has been recognised by governments and policy makers, the economic cost and burden of lending support should not be neglected. A cost-benefit analysis based on individuals’ responses provides an insight into the extent of the incentives required.

## 4 Methods

A CV method was used to evaluate WTP and WTA values. The underlying demand function is the individual’s WTP. In addition, policy implications may be drawn from CV responses which can be used to regulate the extent of the subsidies and other types of financial incentive (Berry et al., 2012). The conventional CV approach can be administered to respondents in different ways, such as open-ended questions, a payment ladder, or as closed-ended single and double-bounded dichotomous choice questions. An open-ended question asks the respondent directly about the maximum amount they would be willing to pay for good *X*. Despite the assumption that the close-ended referenda format is more incentive compatible than open-ended in the hypothetical study (Arrow et al., 1993; Carson and Groves, 2007), lower WTP values have generally been found to be elicited with open-ended questions (Kriström, 1993; Brown et al., 1996). Balistreri et al. (2001) found both open-ended and dichotomous choice questions over estimated auction values and also the expected value for private goods, although the upward bias in open-ended CV was less than that in dichotomous choice CV questions. List and Gallet (2001) suggest that different elicitation mechanisms, including different auction mechanisms, cause disparity in value (relative to a base case Vickrey 2nd price auction).

Lower WTP can be perceived as being due to the larger non-response proportions in an open-ended format, relative to a dichotomous choice format. Protest bias may affect WTP estimates, but this can differ between markets and referenda, and by type of good, making unambiguous rules to deal with protest bids responses difficult to establish (Jorgensen et al., 1999). In addition, sometimes respondents with a lower propensity to meet the expense of the good in question may overstate their WTP in an open-ended question. Carson and Groves (2007) indicated the impossibility of formulating a simple open-ended matching question that tactically corresponds to an incentive compatible binary discrete choice question in an assessment setting, unless the respondents are provided either with a specific price or a device that chooses the cost independent of the individual’s answer. The use of BDM with the open-ended format is said to facilitate the incentive compatibility of the survey setting (Becker et al., 1964; Sudgen, 1999; Carson and Groves, 2007). With this technique, individuals have the incentive to state their maximum WTP truthfully, and the approach would be free of behavioural bias (Horowitz, 2006).

In addition, to control the hypothetical problem of an SP survey, a number of studies have suggested the use of cheap talk to minimise the hypothetical bias effect either in open-ended or close-ended formats (List, 2001; Brown et al., 2003; Carlsson et al., 2011; Carson and Groves, 2011). With an open-ended question, the cheap talk script resulted in decreasing in the quantity of respondents stating a zero WTP; thus hypothetical bias was circumvented, although the average of WTP appeared to increase (Carlsson et al., 2011). Cheap talk, explaining solar panels, energy generated, and benefits, was employed in both the conventional CV treatment, and in the experimental treatment. So any difference between the conventional and experimental treatments should be attributable to the difference between the conventional and experimental treatments only.

Carson and Groves (2011) stated that cheap talk is not a costless technique for non-market valuation if it influences the actions of players in the game. Therefore, the economic value of the difference with and without its use needs to be estimated. The term cheap talk is used in the game theory in an attempt to prevent the dominant strategy in such a way that an individual has no incentive to lie in the game, the so called the equilibrium strategy. This strategy occurs when players share information consistently and in balance with incentives.

To implement a survey with the objective of gathering truthful responses, it is essential for the survey to be designed in accordance with an incentive compatibility format, owing to the high possibility of an individual over-stating or under-stating the value of the good in question: so called strategic behaviour. This may happen when respondents think that a decision will be made based on their evaluation, but that they will not be called upon to pay their stated price. To avert or minimise some of the limitations of the CV method, an incentivised mechanism can be incorporated prior to asking key questions. For instance, an incentive compatible survey can be implemented through the following instruments: a voting system, price auction, lottery auction, games, prize draw, and the selling and buying of items. The information revealed by the respondents’ answers would be the outcome of incentive strategies and the explicit information about the question itself to the respondent. Following studies by Eisenberger and Weber (1995) and Plott and Zeiler (2005), and Chilton et al. (2012), this study also evaluates WTP and WTA via a conventional and an experimental survey of separate samples of respondents. Conventionally, individuals are asked their maximum WTP and minimum WTA. To help respondents gain a better understanding of minimum WTA and maximum WTP concepts, and the potential consequences of over- and under-stating values, an experimental survey with the incentive compatibility was designed and implemented.

In addition, to control for order effects and allow for a between and within subject evaluation, the study was carried out with two groups of respondents with and without the experimental approach. The respondents of one group were individually asked to respond to the open-ended questions without the use of clarification and experimental values. They were required to state their minimum WTA and maximum WTP for solar technology equipment.

The other survey was elicited with the same open-ended question, but prior to that we used the experimental approach. Prior to eliciting values for the solar technology intervention, we administered a practice BDM using a familiar good and with cheap talk, before asking the main question about PV solar panels. In so doing, we were relying on rationality spillover: whether rationality that is induced by a market-like discipline spills over into a non-market setting involving hypothetical choices (see Cherry et al., 2003).

The results of these two settings were compared to determine the role of the incentivised mechanism. The experimental approach aimed to elicit the truthful minimum WTA and maximum WTP responses, which requires beginning with the respondents’ familiarity with the terminologies prior to asking the main WTA and WTP questions. The protocol included firstly, familiarising respondents with the concepts of minimum WTA and maximum WTP and the consequences of untruthful responses; and secondly, asking respondents to state their minimum WTA and maximum WTP for installation of 1kWp micro-generation solar panels on their premises. Finally, respondents provided some socio-economic and demographic information about themselves.

## 5 Experimental

The content of the protocol was supplemented by visual aids, to aid memory and assist the respondents with the questions. The protocol consisted of two sections on WTA and WTP, and the minimum WTA concept was first introduced and practised. Between five to twelve respondents participated in each group session and the participants were incentivised by the opportunity to enter a prize draw for a prize of €10. The practice procedure started with an introductory session on the study’s subject and brief information was given to them about micro-generation solar technology for the residential sector.

The group discussion began by introducing the term ‘reserve price’ as a substitute for the term maximum WTA. Based on other studies, respondents are usually more comfortable with ‘reserve price’ as a term, and these participants were familiarised with the term by discussing the process of selling (600 m^2^) land in an auction. The reserve price was explained as the lowest fixed price (floor price), at which the land would be offered at the auction sale. This was followed by introducing the term ‘external sealed bid’, and also to simplify the meaning of minimum WTA. Respondents were divided into two groups and asked to discuss a ‘reserve price,’ i.e. the minimum price they would accept for a Teddy (which had been given to them beforehand). Then, the reserve price was compared with a predetermined sealed bid in a second price auction mechanism. After comparison between the respondents’ answers and the sealed bids, the question of ‘why it is always best to be truthful’ was discussed. In particular, the experimenter should clarify the possibility of the undesirable consequences of over- or under-stating, i.e. in the case of over-bidding, there is a danger that the vendor keeps the item rather than selling it. Similarly, this is the case of under-bidding when the item sells for less than it is worth. Respondents were given a ‘memory jogger’ to summarise the key concepts, and their answers were recorded in response books.

The subsequent valuation survey was based on individual answers, so it was important that respondents had some experience of deciding their own WTA for an item. Participants were given two tokens for entry to a prize draw. In each of two rounds, participants recorded their ‘reserve price’ or minimum willingness to accept, for selling the token and foregoing entry into the draw. Their reserve price was compared with a sealed bid in an envelope (100 bids ranging from €1 to €10), which had already been randomly selected from a visible box at the front of the room. If their reserve price was lower than, or equal to, this sealed bid they would sell the token, and receive a higher or equivalent sealed bid, but if the reserve price was higher, s/he would not sell the token and be put into the draw.

In the WTP process, contributors were given €2 to spend, €1 in each round, to buy two tickets for entry to a prize draw for €10. In each round, participants’ maximum willingness to pay was recorded in order to buy a token to enter into a new prize draw. Then, after participants were shown a box of chocolates and told that it would be sold, they were asked how much they were willing to pay for it. In other words, the respondents were asked to bid their maximum willingness to pay for the box of chocolates. Before respondents had revealed their maximum WTP amount for the box of chocolates, they were sufficiently familiarised with the potential consequences of over- or under-bidding. In the case of under-bidding when the offered price for the item is less than it is worth, there is a danger of the item not being sold to the buyer, if the vendor decides not to sell for the offered value. Based on the predetermined value or sealed bid price, the respondent’s maximum WTP was evaluated. Respondents had the memory jogger in their hands throughout the practice in the form of their response books.

## 6 Materials and methods

The survey and questionnaire were vetted and approved by Newcastle University Ethics Committee. Informed consent was obtained from each participant prior to their participation in the study.

At the start of the solar technology evaluation questions, respondents were sufficiently practised and experienced for truthful bidding. In addition, respondents were supported by the memory jogger hand-out throughout the micro-generation solar system evaluation. Then, the respondents’ evaluation of the micro-generation solar technology was carried out using the cheap talk script below:

*The process of the discussion that we went through was implemented with the intention of eliciting your truthful responses. We tried to clarify what will be the consequences of overestimating a value to incentivise you to state an amount close to your actual valuation.*

Then, the participants were requested to imagine that the government or private company was offering to install micro-generation solar panels on their properties. An area of 8 m^2^ was considered for the installation of 1 kWp solar panels, including a space allowance for maintenance; with attendant visual amenity impact. Respondents were asked to consider, *their minimum willingness to accept compensation*, for not being permitted to install 1 kWp solar panels.

After the respondents had answered the first question, they were then asked to imagine that a government or private company had offered to install 1 kWp micro-generation solar panels in an area of 8 m^2^ in their property. Respondents were asked *to reveal their maximum willingness to pay.*

Throughout the evaluation, the respondents were supported with memory joggers and were given sufficient explanations and opportunities to ask questions from the moderator. Finally, participants provided some demographic information, and the session finished with the prize draw.

The target population of this study was drawn from a residential sector in Northern Cyprus. The survey was conducted in urban areas including Nicosia, Famagusta and Kyrenia as well as rural regions, including Karpaz and Iskele, Guzelyurt and Lefke. In total, 105 respondents comprised the sample of this study, and they were the decision makers for the household expenditure, regardless of their gender. All the participants were aged above 18 with a mean age of 45. Each experimental session was comprised of five to twelve participants and it ended in one to two hours depending on the size of the group. The sessions were held at different places such as houses, cafes, companies and university.

The sample population for the conventional CV study was 50 respondents, who were interviewed individually. These respondents were not provided with any clarification on terminologies of maximum WTP and minimum WTA prior to being asked the CV WTA and WTP questions. On the other hand, the opportunity to clarify terminologies was provided in the experimental survey, and this study was conducted with 55 respondents in groups of five to twelve.

## 7 Results

In order to compare the WTA/WTP divergences, the WTA/WTP ratios of the conventional and experimental approaches were calculated separately. Table 1 shows the outcome of the conventional approach, where the mean WTA was €15,418 and the mean WTP was €4392. The WTA/WTP ratio was approximately 3.5:1.

**Table 1:** Conventional approach.

N	Variable	Mean	Standard Deviation	Minimum	Maximum
50	WTA	15,418.85	26,821.11	2800	170,000
50	WTP	4392.95	9053.47	700	60,000
	Ratio	3.50990	2.9625		

Values in Euros, 2013 prices.

In addition, to explore the disparity when the highest bids are removed, a sensitivity analysis was carried out (Bateman et al., 2002). Table 2 shows the results of the truncation analysis for conventional approaches. The top 5% of values were trimmed, which resulted in top values of 50 K and 30 K. Therefore, the mean ratio decreased from 3.50:1 to 1.343:1. However, a degree of arbitrariness is incorporated into the approach.

**Table 2:** Truncation analysis for conventional approach.

N	Variable	Mean	Standard Deviation	Minimum	Maximum
46	WTA	10,737.36	18,756.02	2800	50,000
46	WTP	7992.1	20,655.72	700	30,000
	Ratio	1.343	0.9080		

Values in Euros, 2013 prices.

The result of the experimental mechanism is provided in Table 3. This result explicitly illustrates the function of the experimental mechanism, in that the WTA and WTP values have converged. A significant reduction in WTA values resulted in a mean value of €6390. Therefore, the WTA/WTP converged at 1.08:1. Subsequently, the standard deviation values for WTA and WTP from the experimental mechanism were more consistent and had a lower obtained ratio.

**Table 3:** Experimental mechanism.

N	Variable	Mean	Standard Deviation	Minimum	Maximum
55	WTA	6390.11	5196.85	1700	35,000
55	WTP	5913.77	3222.76	2600	18,000
	Ratio	1.080715	1.612		

Values in Euros, 2013 prices.

As reported in Table 4, participants’ WTP increased from 4392 to 5913 Euros, with a WTP_E_/WTP_C_ ratio equal to 1.34, when they were provided with an intuitive understanding of the terminologies. Similarly, respondents’ WTA decreased from 15,418.85 to 6390 Euros with 0.41 WTA_E_/WTA_C_ ratio.

**Table 4:** Means of WTPs and WTAs.

Variable	Mean WTP	Mean WTA
Experimental-maximum WTP	5913.77 (3222.76)	6390.11 (5196.85)
Conventional-maximum WTP	4392.95 (9053.47)	15,418.85 (26,821.11)
Ratio	1.34 (0.36)	0.41 (0.19)

Values in Euros, 2013 prices, Standard deviation in parenthesis.

Additionally, a *T* test shows that the difference between WTA_E_–WTA_C_ is statistically significant at the 0.05 level, whereas the difference between WTP_E_–WTP_C_ is not statistically significant.

Nevertheless, it is noteworthy that the WTA_E_ value was considerably influenced by the impact of the experimental setting compared with the WTP_E_ value. The significant reduction in WTA_E_ values via experimental setting implies that there is a greater need for clarification on WTA term compared with WTP. In other words, it is more important to tackle the elicitation of truthful responses from WTA questions than from WTP questions.

Finally, a *T* test on the difference between WTA_E_–WTP_E_ is insignificant, whereas it is significant between WTA_C_–WTP_C_

As a result, the experimental approach showed a lower ratio (WTA/WTP) than has previously been reported in the environmental economics literature. The average WTA value was significantly influenced by the incentivised setting and its value sharply decreased, whereas the average value of WTP was not substantially greater than in conventional studies.

## 8 Discussion

This study tested the role of incentives on individuals’ estimation of WTA and WTP for micro-generation solar panels. The discrepancies between WTA and WTP valuations are recognised as an obvious problem in the CV surveys. However, true preferences can be elicited through an incentivised mechanism. The incentive-compatible mechanism provides respondents with an adequate understanding and does not encourage strategic biases (Sudgen, 1999). The reduced discrepancy between the conventional and experimental mechanisms agrees with the economic theory and literature findings.

The suggested novel experimental approach allowed the convergence of WTA and WTP, when the respondents were sufficiently incentivised to respond. The average discrepancy based on the 45 studies on WTA/WTP ratio was found by Horowitz and McConnell (2002) to be (10.4:1) for public and non-market goods, with a ratio of 2.9:1 for ordinary private goods. The conventional setting results here, with an average WTA/WTP 3.5:1 ratio is consistent with the average ratio in the literature. This ratio substantially decreased to 1.08:1 in the experimental or incentivised setting. Consequently, this finding agrees with the hypothesis that the incentivised setting will perform better than the conventional setting in terms of avoiding strategic and hypothetical biases. The perceived larger sum to compensate in the conventional setting corroborates previous studies (Knetsch and Sinden, 1984).

The findings agree with studies by Scarpa and Willis (2010) and Claudy et al. (2011) on WTP for micro-generation, in that households are willing to pay for micro-generation systems, but the benefit households receive from micro-generation are not sufficiently large to cover the capital cost of micro-generation energy technologies. Financial incentives are thus required to encourage people to invest in micro-generation technologies, if renewable energy targets are to be met. However, the findings of the suggested novel experimental setting here indicate a higher support from respondents for covering the capital costs of micro-generation solar technology. This was achieved when individuals had a better understanding of the WTA and WTP questions, the consequences of overestimating and underestimating, and the good in question (micro-generation solar technology). Subsequently, they revealed truthful responses.

## 9 Conclusions

This paper assesses the households’ acceptance and preferences for the installation of micro-generation solar panels in the residential sector. The individuals’ WTA compensation for the loss of a 1 kWp solar panel (i.e. loss of electricity generated for own personal consumption and export to the grid), and WTP for installation of 1 kWp solar panel, was tested. The survey was implemented via conventional and incentivised settings. The discrepancy between WTA and WTP within each setting and between the settings was compared. The most obvious findings are: (1) that WTA is statistically different to WTP in the conventional setting, whereas it is equivalent in the experimental setting; (2) a smaller value of WTA for compensation and larger WTP are observed in the incentivised setting compared with the conventional setting.

Conventional CV methods may not derive truthful WTA and WTP responses. The experimental setting results suggest that policy makers could reduce financial incentives to increase the solar power installations in Cyprus.

## Declarations

### Author contribution statement

Mehrshad Radmehr: Conceived and designed the experiments; Performed the experiments; Analyzed and interpreted the data; Contributed reagents, materials, analysis tools or data; Wrote the paper.

Ken Willis: Analyzed and interpreted the data; Contributed reagents, materials, analysis tools or data; Wrote the paper.

Hugh Metcalf: Conceived and designed the experiments; Analyzed and interpreted the data; Contributed reagents, materials, analysis tools or data; Wrote the paper.

### Competing interest statement

The authors declare no conflict of interest.

### Funding statement

This research did not receive any specific grant from funding agencies in the public, commercial, or not-for-profit sectors.

### Additional information

No additional information is available for this paper.
